# Waste management in disease prevention: A public health overview

**DOI:** 10.6026/973206300214363

**Published:** 2025-12-15

**Authors:** Meghna Dipakkumar Chaudhary, Rajvi D Chaudhary, Kshitij Sale, Chaitanya Chaudhary, Uma Vijayashankar, Pranav Manek, Miral Mehta, Dhaval Niranjan Mehta

**Affiliations:** 1 Department of Environmental Health & Safety, Amneal Pharmaceuticals, USA; 2 Health Informatics, Rutgers University, USA; 3 Alumni, Information Technology, Rutgers University, USA; 4 Artificial Intelligence & Machine Learning, Rutgers University, USA; 5 Department of Physiology, JSS Medical College, JSS Academy of Higher Education & Research, Mysore, Karnataka, India; 6 Department of Global and Population Health, Henry M Goldman School of Dental Medicine, Boston University, USA; 7 Department of Pediatric and Preventive Dentistry, Karnavati School of Dentistry, Gujarat, India; 8 Department of Oral Medicine and Radiology, Narsinhbhai Patel Dental College and Hospital, Sankalchand Patel University, Visnagar, Gujarat, India

**Keywords:** Waste management, public health, disease prevention, sanitation, environmental health, infectious diseases, vector-borne diseases

## Abstract

The rapid rise in urbanization and consumption has intensified global solid waste generation, creating pressing public health challenges. Improper waste disposal contributes to infectious diseases via vector proliferation, water and soil contamination and direct exposure to hazardous materials. This review synthesizes evidence on health risks from municipal, healthcare and electronic waste while evaluating interventions within the integrated solid waste management framework. Data shows the link between poor waste management and increased incidence of vector-borne, waterborne, respiratory and chronic diseases. An integrated, multi-sectoral approach grounded in circular economy principles is essential to safeguard human and environmental health.

## Background:

The global production of municipal solid waste (MSW) is staggering, estimated at over two billion tonnes annually and projected to increase by 70% by 2050, largely driven by rapid urbanization and economic development [1]. While waste is an inevitable byproduct of human activity, its management is a critical determinant of public health outcomes. Historically, the link between filth and disease has been a cornerstone of public health, with the sanitary reforms of the 19th century dramatically reducing mortality from infectious diseases in industrializing cities [2]. Today, despite significant advancements, billions of people, predominantly in low- and middle-income countries (LMICs), still lack access to basic waste collection services, resulting in open dumping, uncontrolled burning and widespread environmental contamination [3]. This failure in waste management creates a complex web of health hazards. The accumulation of organic waste provides fertile breeding grounds for pathogenic microorganisms and disease vectors, while hazardous components from healthcare, industrial and electronic sources introduce toxic substances into the environment [4]. The significance of this issue extends beyond communicable diseases; chronic exposure to pollutants from waste is linked to respiratory illnesses, developmental disorders and certain cancers [5]. Current knowledge firmly establishes correlations between poor waste management and adverse health outcomes. However, a significant controversy or challenge in the field is the difficulty in establishing direct causal links and quantifying the precise burden of disease attributable to waste, due to confounding factors like socioeconomic status, hygiene practices and healthcare access [6]. Furthermore, a gap exists between the recognition of the problem and the implementation of effective, sustainable solutions, particularly in resource-constrained settings. Debates continue regarding the optimal balance between high-tech, capital-intensive solutions (e.g., waste-to-energy incineration) and low-cost, community-based models. Specifically, this review will: (1) detail the epidemiological pathways linking waste to disease; (2) examine the distinct risks posed by different waste streams; (3) critically evaluate the hierarchy of public health interventions in waste management; and (4) discuss the challenges and future directions for integrating waste management into mainstream public health policy and practice. Therefore, it is of interest to comprehensively describe the public health implications of waste management.

## Review:

## Pathophysiological pathways of disease transmission from inadequate waste management:

The mechanisms through which mismanaged waste contributes to disease are diverse and interconnected, operating through biological, chemical and physical vectors.

## Vector proliferation and vector-borne diseases:

Uncollected solid waste serves as an ideal habitat and nutrient source for a wide range of disease vectors. Open dumpsites, water-filled containers like discarded tires and plastics and blocked drainage channels filled with waste create prolific breeding sites for mosquitoes [7]. The **Aedes* aegypti* mosquito, the primary vector for dengue, chikungunya, Zika and yellow fever, thrives in such artificial water containers common in urban waste [8]. Studies from Brazil and India have demonstrated a strong spatial correlation between high densities of MSW and increased larval indices for *Aedes*, directly linking waste accumulation to higher risks of dengue outbreaks [9, 10]. Similarly, stagnant, polluted water pools resulting from waste blockages are breeding grounds for *Culex* mosquitoes, vectors of West Nile Virus and lymphatic filariasis and Anopheles mosquitoes, responsible for malaria transmission in some peri-urban settings [11]. Beyond mosquitoes, decomposing organic waste attracts rodents (rats, mice) and insects (flies, cockroaches). Rodents can transmit numerous pathogens, causing diseases such as leptospirosis, Hantavirus pulmonary syndrome and salmonellosis, while their fleas can carry the plague bacterium, *Yersinia pestis* [12]. Mechanical vectors like houseflies (*Musca domestica*) breed in refuse and can physically transfer pathogens like *Shigella*, *Salmonella* and *E. coli* from faecal matter in waste to human food, causing diarrhoeal diseases [13].

## Contamination of water, soil and food:

When waste is disposed of in uncontrolled dumps or unlined landfills, it generates a highly toxic liquid known as leachate. This leachate, containing dissolved organic and inorganic compounds, heavy metals (e.g., lead, cadmium, mercury) and pathogenic microorganisms, can percolate through the soil and contaminate groundwater aquifers, which are often sources of drinking water [14]. The ingestion of water contaminated with pathogens from human and animal excreta in waste is a primary route for waterborne diseases such as cholera, typhoid fever, giardiasis and hepatitis A and E [15]. Chemical contamination of drinking water with heavy metals and persistent organic pollutants from waste can lead to chronic health effects, including renal dysfunction, neurological damage and cancer, over long-term exposure [5]. Soil contamination also affects the food chain, as crops grown in polluted soil can bioaccumulate heavy metals, posing a risk to human consumption.

## Direct contact and occupational hazards:

Direct contact with waste poses significant health risks, particularly for vulnerable populations such as children playing near dumpsites and the millions of informal waste pickers who segregate and recover recyclable materials for their livelihood [16]. These individuals face high risks of physical injuries (e.g., cuts from sharp objects) leading to infections like tetanus. They are also exposed to a myriad of pathogens, resulting in a high prevalence of dermal infections, respiratory problems and gastrointestinal illnesses [17]. The lack of personal protective equipment (PPE) exacerbates these risks.

## Air pollution from waste decomposition and burning:

The anaerobic decomposition of organic waste in landfills is a major anthropogenic source of methane, a potent greenhouse gas and other malodorous compounds like hydrogen sulfide. More acutely, the common practice of open waste burning in areas lacking collection services releases a cocktail of hazardous air pollutants, including particulate matter (PM2.5), dioxins, furans and volatile organic compounds [18]. Inhalation of these substances is strongly associated with acute respiratory infections, aggravation of asthma and chronic obstructive pulmonary disease (COPD) and an increased long-term risk of cardiovascular disease and lung cancer [19].

## Specific waste streams and associated health risks:

While all mismanaged waste is hazardous, certain streams present unique and severe public health challenges.

## Municipal solid waste (MSW):

MSW is the most voluminous waste stream and is responsible for the majority of the vector breeding and environmental contamination issues discussed above. Its heterogeneous nature, containing everything from food scraps to plastics and hazardous household items, makes its management complex. The high organic fraction in the MSW of many LMICs (often >50%) makes it particularly prone to rapid decomposition, odour generation and vector attraction [1].

## Healthcare waste (HCW):

Generated by hospitals, clinics and laboratories, HCW is of grave concern due to its infectious and hazardous nature. It includes "sharps" (needles, scalpels), infectious waste (cultures, blood-soaked materials), pathological waste and pharmaceutical waste. Improper disposal of HCW, such as mixing it with MSW, exposes healthcare workers, waste handlers and the general public to significant risks. Sharps injuries can transmit blood-borne pathogens, including Human Immunodeficiency Virus (HIV), Hepatitis B Virus (HBV) and Hepatitis C Virus (HCV) [20]. A WHO report estimated that in 2000 alone, injections with contaminated syringes caused 21 million HBV infections and 260,000 HIV infections globally [21, 22].

## Electronic waste (e-waste):

The exponential growth of consumer electronics has created a tidal wave of e-waste, which contains valuable but also highly toxic materials. In many LMICs, e-waste is processed in the informal sector using primitive methods like open burning of cables to recover copper and acid leaching to extract gold [23]. These practices release heavy metals such as lead, mercury, cadmium and brominated flame retardants into the air, soil and water. Exposure to these neurotoxins is particularly damaging to children, whose developing nervous systems are highly vulnerable. Studies conducted in e-waste recycling sites in China and Ghana have documented elevated blood lead levels in children, associated with reduced IQ scores, behavioural problems and other developmental deficits [24, 25].

## Public health interventions: An integrated approach:

Effective disease prevention requires a multi-pronged strategy for waste management, often conceptualized as the Integrated Solid Waste Management (ISWM) hierarchy. This model prioritizes waste prevention and reduction, followed by reuse, recycling, recovery and finally, environmentally sound disposal.

## Source reduction and segregation:

The most effective public health intervention is to prevent waste generation at its source. Policies promoting sustainable consumption, eco-design and extended producer responsibility can significantly reduce the volume and toxicity of waste [26]. At the household and institutional level, segregation of waste into distinct streams (e.g., organic, recyclable, hazardous) is a critical first step. In healthcare settings, meticulous segregation of infectious waste from general waste is paramount for preventing nosocomial and community-based infections. This requires continuous training of staff, clear labelling and the provision of appropriate, puncture-proof containers [27].

## Collection, transportation and formalization:

Regular and reliable waste collection is the backbone of any functional system. It prevents the accumulation of waste in residential areas, thereby eliminating vector breeding sites and reducing direct human contact. For this to be effective, collection systems must be comprehensive, covering both affluent and low-income neighbourhoods. A key intervention in many cities is the formalization and integration of the informal recycling sector. By providing waste pickers with legal recognition, fair wages, safety training, PPE and access to healthcare (including vaccinations for tetanus and hepatitis B), cities can improve both the efficiency of recycling and the health and dignity of these essential workers [28].

## Treatment and Disposal Technologies:

## Sanitary landfills:

In contrast to open dumps, modern sanitary landfills are engineered containment facilities designed to minimize environmental impact. Key features include impermeable liners to prevent leachate contamination of groundwater, leachate collection and treatment systems and gas collection systems to capture methane for energy use or flaring [29]. While a significant improvement, they are land-intensive and require long-term monitoring.

## Composting and anaerobic digestion:

These biological treatment methods are ideal for the organic fraction of MSW. Composting produces a soil conditioner, while anaerobic digestion produces biogas (a renewable energy source) and a nutrient-rich digestate. By diverting organic waste from landfills, these technologies reduce methane emissions, decrease leachate production and limit vector proliferation [30].

## Incineration (Waste-to-Energy):

Incineration involves the controlled combustion of waste, which dramatically reduces its volume and can be used to generate electricity or heat. Modern incinerators are equipped with advanced flue gas cleaning systems to control the emission of pollutants like dioxins and heavy metals [18]. However, they are capital-intensive to build and operate, require a consistent supply of high-calorific-value waste and face public opposition due to perceived air pollution risks. For HCW, high-temperature incineration or alternative technologies like autoclaving are considered the gold standard for sterilizing infectious materials [20].

## Discussion:

The synthesized evidence overwhelmingly confirms that waste management is not merely an aesthetic or environmental service but a core component of public health infrastructure. The pathways from unmanaged waste to disease are direct, multifaceted and disproportionately affect the most vulnerable populations. Interventions based on the ISWM hierarchy offer a clear roadmap for mitigating these risks. However, the translation of this knowledge into practice is fraught with challenges. A significant gap in the literature is the scarcity of robust, longitudinal studies that quantify the reduction in disease incidence following the implementation of specific waste management interventions. Most studies are cross-sectional and demonstrate correlation rather than causation, making it difficult to isolate the impact of waste management from other concurrent improvements in sanitation, water supply and socioeconomic conditions [6]. More rigorous quasi-experimental and controlled studies are needed to build a stronger evidence base for policymakers and justify investments. Furthermore, much of the research focuses on the infectious disease risks of MSW, while the long-term health impacts of chronic chemical exposure from e-waste and industrial waste in LMICs remain under-investigated. The mental health consequences for residents living in proximity to large dumpsites-including stress, depression and reduced quality of life due to odour, pests and social stigma-is another critical area requiring further research. The implications of this review are clear. For policymakers, waste management must be reframed as a public health imperative and integrated into national health strategies and urban planning. This requires shifting from a reactive, end-of-pipe approach to a proactive, systems-based one that embraces the circular economy-designing waste out of the system. This entails enforcing regulations, allocating sustainable financing and building institutional capacity. For practitioners, a "one-size-fits-all" approach is ineffective. Solutions must be context-specific, considering local socioeconomic conditions, waste composition and cultural practices. Community participation is not optional; it is essential for the success of initiatives like source segregation and local composting [31]. Protecting the health of sanitation and recycling workers through occupational health and safety programs must be a non-negotiable priority. Finally, the research community has a responsibility to conduct interdisciplinary research that bridges engineering, public health, economics and social sciences to develop and evaluate cost-effective, sustainable and socially equitable waste management models. Emerging threats, such as the role of plastic waste in creating new vector habitats and the dissemination of AMR genes and microplastics through waste streams, demand urgent scientific attention [22].

## Conclusion:

Waste management is a fundamental determinant of human health and a critical tool for disease prevention. The failure to properly contain and treat waste unleashes a cascade of public health threats, from fostering epidemics of vector-borne and waterborne diseases to causing chronic illness through long-term exposure to toxic pollutants. The evidence demonstrates that a well-designed ISWM system-prioritizing reduction, segregation, recycling and safe disposal-can effectively sever these transmission pathways.
